# Obstetric complications in pregnant people with Fontan circulation: Identifying candidate pathologic pathways in the placenta

**DOI:** 10.1002/pmf2.70091

**Published:** 2025-09-08

**Authors:** Ashley Hesson, Alexa Pinsky, Angela Quain, Caroline Simon, Levi J. Anderson, Michael Joynt, Elizabeth Langen

**Affiliations:** ^1^ Division of Maternal Fetal Medicine Department of Obstetrics and Gynecology University of Michigan Ann Arbor Michigan USA; ^2^ Division of Pediatric Cardiology, Department of Pediatrics University of Michigan Ann Arbor Michigan USA; ^3^ Department of Cardiology, Frankel Cardiovascular Center University of Michigan Adult Congenital Heart Disease Program Ann Arbor Michigan USA; ^4^ Department of Pathology University of Michigan Ann Arbor Michigan USA

## Abstract

**Background/Purpose:**

Pregnant people with Fontan circulation have high rates of fetal growth restriction (FGR) and preterm birth (PTB). The mechanisms underlying these complications are poorly understood.

**Methods:**

Pregnancies occurring in individuals with Fontan circulation cared for in a single Cardio‐Obstetrics program were identified. Demographic and clinical data were abstracted from the electronic medical record. A subset of FGR and PTB placental samples was processed for histopathology evaluation. Regions with dense representation of pathology were selected for sequencing by a placental pathologist. Sequencing was performed on the Visium CytAssist platform, with analysis in Loupe Browser and R Studio. Gene Ontologies were generated using the DAVID analytic suite.

**Results:**

A total of 21 placentas from pregnancies complicated by maternal Fontan circulation were identified and six were selected to undergo spatial transcriptomic analysis. FGR and PTB prior to 34 weeks gestation were represented in half of the placentas (3/6, respectively); the remaining three placentas represented gestational age‐appropriate fetal growth. The most common histopathologic findings were subchorionic hematomas (13/21) and accelerated maturation (12/21). Decidual arteriopathy and features of fetal vascular malperfusion were rarely observed (1/21 and 4/21). Comparing the spatially aligned RNA expression spots from the placentas with and without FGR, 1473 genes were differentially expressed (*p* < 0.05). Functional clustering of the top 100 differentially expressed genes revealed alterations in stress‐related hormones/transcription factors and pregnancy‐modulating hormone pathways. These markers of endocrinologic stress and shallow placentation were overexpressed by synciotrophoblasts. PAPPA and the PSG family of transcripts were upregulated in FGR and spontaneous PTB cases; these transcripts have been previously associated with placental hypoxia.

**Conclusion:**

Comparative spatial transcriptomic analysis of placentas from individuals with Fontan circulation who did and did not experience major obstetric complications identified upregulation of markers associated with poor placentation and endocrinologic stress.

## 1 INTRODUCTION

Among pregnancies complicated by congenital heart disease, those with single‐ventricle physiology have the highest complication rate [1]. Common adverse outcomes include spontaneous abortion, spontaneous preterm birth, fetal growth restriction, and peripartum hemorrhage [2]. Larger series report live birth rates under 50% [3]. These events do not correlate with known indicators of decompensation in this population [2]. As surgical repairs of the diverse congenital lesions undergoing single‐ventricle repairs improve, the number of individuals with single‐ventricle physiology in pregnancy is increasing [4]. Thus, there is increasing urgency to better understand the mechanisms and predictors of obstetric morbidity in this growing subset of the Cardio‐Obstetric patient base.

The modern standard for single‐ventricle palliation is a series of surgeries culminating in the Fontan operation. First designed in 1971, the Fontan operation creates a passive pulmonary circuit, wherein venous return flows through a cavopulmonary shunt. This bypasses one of the two ventricles, repairing lesions such as hypoplastic left heart syndrome and pulmonary atresia. The remaining ventricle, of either left or right morphology, becomes the systemic pumping chamber [5]. Though the Fontan operation has improved outcomes compared to prior management—enhancing the likelihood of survival through reproductive age—Fontan physiology is marked by life‐long elevated systemic venous pressures, chronic hypoxemia and reduced cardiac output, which has consequences for nearly every major organ system [6, 7].

Prior work describing placental pathology in individuals with Fontan circulation highlighted the consistent presence of prominent subchorionic fibrin deposition. This finding was sometimes associated with an abnormal circumferential fetal membrane insertion. In addition to preeclampsia and hypoxemia, Fontan physiology may consistently lead to substantial venous resistance, chronic small abruptions, and stagnation of placental blood flow. Although not significantly displacing or entrapping the villi, subchorionic fibrin might underperfuse the fetus by compressing the chorionic and larger stem vessels, potentially contributing to fetal growth restriction [8]. Another small cohort study noted a potential relationship between ultrasound findings and post‐placental bleeding. Ultrasonographic examination revealed placental edematous changes in 8 of the 15 pregnancies studied (53%) [9]. Subchorionic hemorrhage was noted in five pregnancies within the placental edematous change group, whereas none were observed in the group without such changes [9].

Though extracardiac effects range from protein‐losing enteropathies to endocrinopathies and impaired fertility [7], Fontan‐associated liver disease (FALD) is among the more profound and well‐described extra‐cardiac complications of single‐ventricle physiology. FALD is a distinct form of fibrotic liver disease with a hypothesized mechanism related to chronically elevated, non‐pulsatile central venous pressures. A combined histopathologic and multi‐omics approach has previously been applied to liver biopsies from patients with Fontan palliations to understand the mechanism underlying this pathology, demonstrating derangements in amino acid metabolism, glycolysis, and gluconeogenesis [10]. Single‐cell transcriptomics identified metabolic stress as well, but also highlighted alterations in cell–cell adhesions and anti/pro‐angiogenic balance [11]. A similar approach has been applied in disease states with known placental effects and recent studies have demonstrated the vast landscape of data that can be mined from placental tissues at the RNA level [12, 13]. Though case series have suggested the presence of widespread placental pathology in the setting of single‐ventricle physiology [14], there have yet to be any reported biomolecular evaluations of placentas from patients with Fontan circulation.

In this study, we aimed to elucidate the pathological pathways in placentas of patients with Fontan circulation using spatial transcriptomics. We hypothesized that much like other Fontan‐associated disease states, there would be molecular signatures of vascular dysfunction and metabolic stress. We explore transcriptional targets and pathways using histopathologically co‐located RNA sequencing from placentas from patients with Fontan physiology. The results of this hypothesis‐generating study will establish the first spatial transcriptomic dataset of placentas from patients with Fontan circulation and contribute exploratory mechanistic insights into the obstetric morbidity observed in this population.

## 2 METHODS

### 2.1 Sampling

Pregnancies occurring in individuals with Fontan circulation cared for in a single Cardio‐Obstetrics Program from 2000 to 2024 that resulted in a live birth were included. Demographic and clinical data were abstracted from the electronic medical record. Placental samples stored from these pregnancies as formalin‐fixed, paraffin‐embedded blocks were obtained for histopathologic re‐evaluation. The subset of placental samples selected was chosen based on the presence or absence of fetal growth restriction (FGR) and gestational age at delivery. This work was determined to be exempt by the study site's institutional review board (HUM00244157).

A pre‐determined sample size of six cases was specified for transcriptomic analysis. We selected three placentas of pregnancies complicated by FGR defined as an estimated fetal weight of less than the 10th percentile at any point in pregnancy and three samples from pregnancies with normal fetal growth for gestational age. Equal sampling of biologic replicates across the key clinical comparison was performed to allow for assessment of differential expression within samples (pathologic vs. non‐pathologic regions) while ensuring consistency between clinically grouped placental specimens. All of the selected placentas were from preterm births (less than 37 completed weeks of gestation). Births occurring at fewer and more than 34 completed weeks had equal representation within spatial transcriptomics subsample (*N* = 3, respectively). This sampling was intended to represent the preterm skew of the clinical sample.

### 2.2 Materials and procedures

**FIGURE 1:**
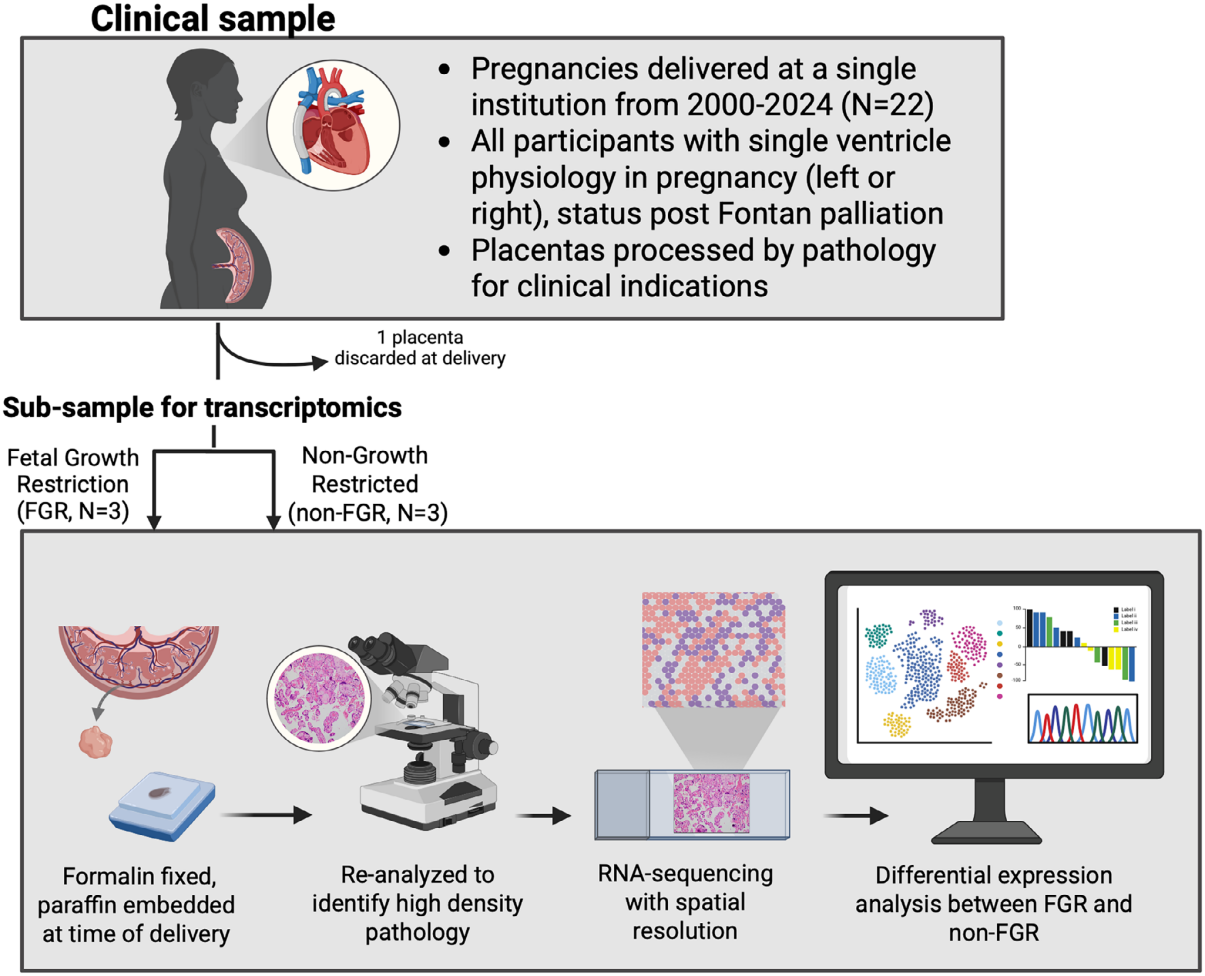
Overview of study methods.

Figure 1 summarizes the methods pertaining to placental analysis. The analyzed placentas were initially preserved per the standard clinical pathology protocol following delivery. The placentas for this study were re‐analyzed by a subspecialized placental pathologist (part of the authorship team), who identified representative histopathologic findings and areas of high‐density pathology on hematoxylin and eosin‐stained sections. The specific pathologic findings are detailed in the Results, but include areas of subchorionic hemorrhage, syncytial knotting, and loss of typical architecture within the villi. These areas were selected to optimize application of the spatial transcriptomic technique, where differential expression analysis is co‐located with histopathology. Paraffin scrolls were created of the selected areas and processed into Visium slides with a 6.5 mm × 6.5 mm capture area. A total of 5000 spots per capture area were acquired. Per sample, 90 million paired and 180 million total reads per sample were processed. RNA extraction, spatial transcriptomic library preparation, and sequencing were performed by MedGenome, Inc. (Foster City, CA) using the Visium CytAssist platform (10x Genomics; Pleasanton, CA). The sequencing data are available in the Gene Expression Omnibus (GEO) under accession number GSE303933. Initial processing and quality assessment were conducted in SpaceRanger (10xGenomics), with preliminary analyses and spot selection in Loupe Browser (10x Genomics Loupe Browser v8.0.0). Spots that were associated with acellular areas or poor RNA quality, assessed via a combination of visual inspection and expression markers, were excluded prior to differential expression analysis. Unsupervised clustering was performed and noted to correlate with the pre‐defined clinical groups, which were accordingly carried forward in the analysis. Differential expression analysis was performed natively in Loupe Browser. Transcripts with an adjusted p‐value of < 0.05 across the primary comparison of growth restricted (FGR) versus size appropriate for assigned gestation age (non‐FGR) spots were considered to be significantly differentially expressed.

Additional analyses and visualizations of the significantly differentially expressed transcripts as well as descriptive statistics and hypothesis testing of the demographic/ clinical data were conducted using R Statistical Software [15] with the R Studio (v2024.12.0+467) wrap [16]. Gene ontologies were generated using the DAVID analytic suite [17, 18] utilizing Geoterm and Kegg ontology sets. Only hits with an enrichment score of > 1 and *p* values < 0.05 were retained. Additional automated functional classification was performed using gene set enrichment analysis with the EnrichR package within R [19], applying the Bioplanet 2019 library. Given the highly specific context of placental pathology (that is underrepresented in generalized gene ontologies) the top 50 upregulated and downregulated genes in FGR (as compared to non‐FGR) placentas were manually reviewed and annotated using summative resources [20] and the available literature. Initial categorizations were made by two authors, then reviewed for consistency and parsimony by another author.

## 3 RESULTS

A total of 22 pregnancies resulting in a live birth for people with Fontan circulation were included. These pregnancies occurred in 17 people, where 4 people had multiple pregnancies over the course of the study period. Of the studied pregnancies, placentas were collected for pathologic exam from 21 deliveries; one placenta was discarded shortly after birth.

### 3.1 Sample characteristics and pregnancy outcomes

Table 1 summarizes the demographic and clinical composition of the pregnancies studied. The average age at the time of delivery was 25.8 ± 4.3 years. Hypoplastic left heart syndrome (HLHS) was the most common congenital lesion for which a Fontan palliation was performed (36.4% of the sample, *N* = 8/22). All patients had New York Heart Association class 1 or 2 functional status pre‐pregnancy, and the vast majority had a normally functioning single ventricle (86.4%, 19/22). The mean pre‐pregnancy oxygen saturation was 93.6% ± 3.3 in the sample.

**TABLE 1:** **Demographic characteristics**. Precents and *N*s versus means and standard deviations given as appropriate.

Parameter	Value
**Age (years)**	25.8 ± 4.3
**BMI**	30.7 ± 5.2
**Race**	
Caucasian	59.1% (13/22)
Hispanic	9.1% (2/22)
Black	27.3% (6/22)
Multi‐racial	4.5% (1/22)
**Diagnosis**	
HLHS	36.4% (8/22)
Other	63.6% (14/22)
**Ventricular morphology**	
Left	54.5% (12/22)
Right	45.5% (10/22)
**NYHA class**	
1	84.2% (16/19)
2	15.8% (3/19)
**Pre‐pregnancy oxygen saturation (%)**	93.6 ± 3.3
**Pre‐pregnancy ventricular function**	
Normal	86.4% (19/22)
Mildly depressed	9.1% (2/22)
Severely depressed	4.5% (1/22)

Abbreviations: BMI, body mass index; HLHS, hypoplastic left heart syndrome; NYHA, New York Heart Association.

The mean gestational age at delivery across identified pregnancies was 237.8 ± 23.3, equating to 33 weeks and 6 days of completed gestation. Nearly half of the sample 45.5 % (*N* = 10/22) delivered by cesarean birth, while spontaneous and operative vaginal births were equally represented in the remainder. Over 40% (*N* = 9/22) of the pregnancies were diagnosed with fetal growth restriction and the majority (59.1%, *N* = 13/22) of neonates born in the sample required admission to neonatal intensive care upon delivery. See Table 2 for additional pregnancy outcomes for the studied cohort. The cases specifically included in the spatial transcriptomic analysis are summarized individually in Table 3.

**TABLE 2:** Pregnancy outcomes. Precents and *N*s versus means and standard deviations given as appropriate.

Parameter	Value
**Gestational age at delivery (days)**	237.8 ± 23.3
**Fetal growth restriction**	
Yes	40.9% (9/22)
No	59.1% (13/22)
**Delivery mode**	
Cesarean	45.5 % (10/22)
Spontaneous vaginal	27.3% (6/22)
Operative vaginal	27.3% (6/22)
**NICU admission**	
Yes	59.1% (13/22)
No	40.9% (9/22)

Abbreviation: NICU, neonatal intensive care unit.

**TABLE 3:** Pregnancy and placental characteristics of samples submitted for spatial transcriptomics.

Cases selected for transcriptomic analysis															
Case #	Gestational age	FGR	Subchorionic hematoma present	Infarcts	Retroplacental Hemorrhage	Distal villous hypoplasia	Increased syncytial knotting	Accelerated maturation for gestational age	Decidual arteriopathy	Thrombosis	Avascular villi	Intramural fibrin deposition	Villous stromal vascular Karyorrhexis	Stem vessel obliteration	High Grade vs low grade
1	30w4d	Y	Y	N	Y	N	N	N	N	N	N	N	N	N	N/A
2	30w3d	Y	Y	N	N	Y	Y	Y	N	N	Y	Y	Y	Y	HG
3	35w1d	Y	Y	Y	Y	Y	Y	Y	N	N	N	N	N	N	N/A
4	36w0d	N	Y	N	N	N	N	N	N	N	N	N	N	N	N/A
5	30w6d	N	Y	N	N	Y	Y	Y	N	N	N	N	N	N	N/A
6	34w3d	N	N	N	N	N	N	N	Y	N	Y	N	N	N	LG

Abbreviations: HG, high grade; LG, low grade; N, no; NA, not applicable; Y, yes.

### 3.2 Histopathologic findings

**FIGURE 2:**
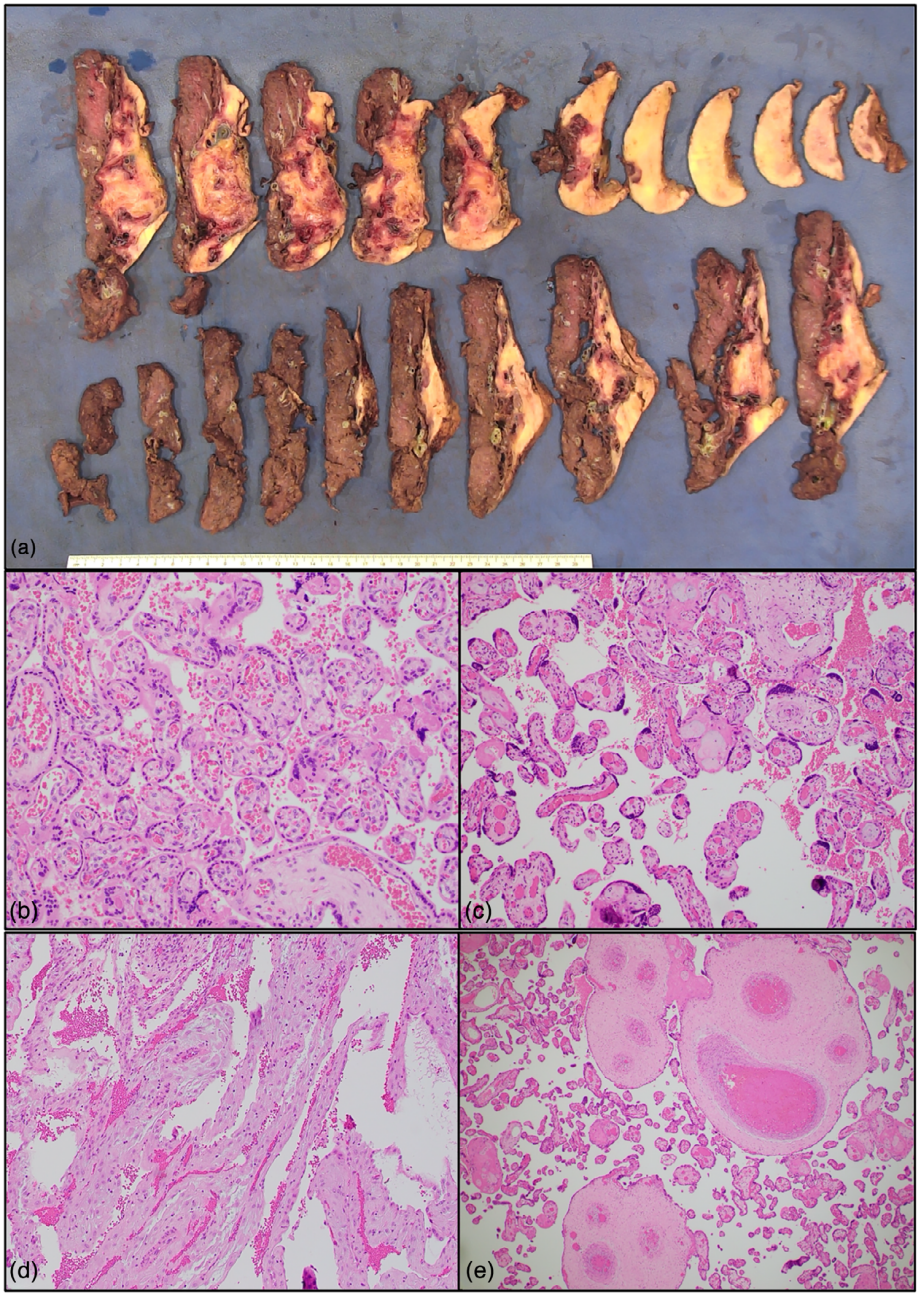
Gross and microscopic features of placentas from patients with Fontan circulation. (A) Subchorionic bleed shown in a grossed specimen (pale, organized clot). (B) Appropriate villous maturation for gestational age [H&E 2000078]. (C) Increased syncytial knotting and distal villous hypoplasia, consistent with accelerated maturation for gestational age [H&E 200×]. (D) Fetal vascular malperfusion [H&E 100×]), (E) High‐grade fetal vascular malperfusion [H&E 40×].

Overall, the *N* = 21 examined placentas had extensive evidence of gross and microscopic pathology. Figure 2 illustrates an area of normal appearing placental parenchyma (panel B), compared to abnormal findings on microscopic (panels C–E) and gross (panel A) exam. Grossly, the weight of the placentas varied widely (from < 3 to 95–97th percentile) with a mean weight of 383.5 ± 179.1 g. The most common histopathologic feature was subchorionic hematoma, which was seen in 61.9% (*N* = 13/21) of cases. Accelerated maturation for gestational age, evinced by syncytial knotting and distal villous hyperplasia, was present in 57.1% (*N* = 12/21) specimens. Decidual arteriopathy and features of fetal vascular malperfusion were observed in a minority of cases (4.7% [*N* = 1/21] and 19.0% [*N* = 4/21], respectively). The findings for the cases that were processed for spatial transcriptomics are summarized alongside their clinical data in Table 3.

### 3.3 Spatial transcriptomics

The subset of placentas selected for spatial transcriptomic analysis (*N* = 3 from pregnancies diagnosed with FGR and *N* = 3 well‐grown cases) was reviewed for the aforementioned histopathologic features that were characteristic of the overall set. The specific sections that were submitted for spatial transcriptomic processing and analysis had evidence of all of these features (Table 3).

**FIGURE 3:**
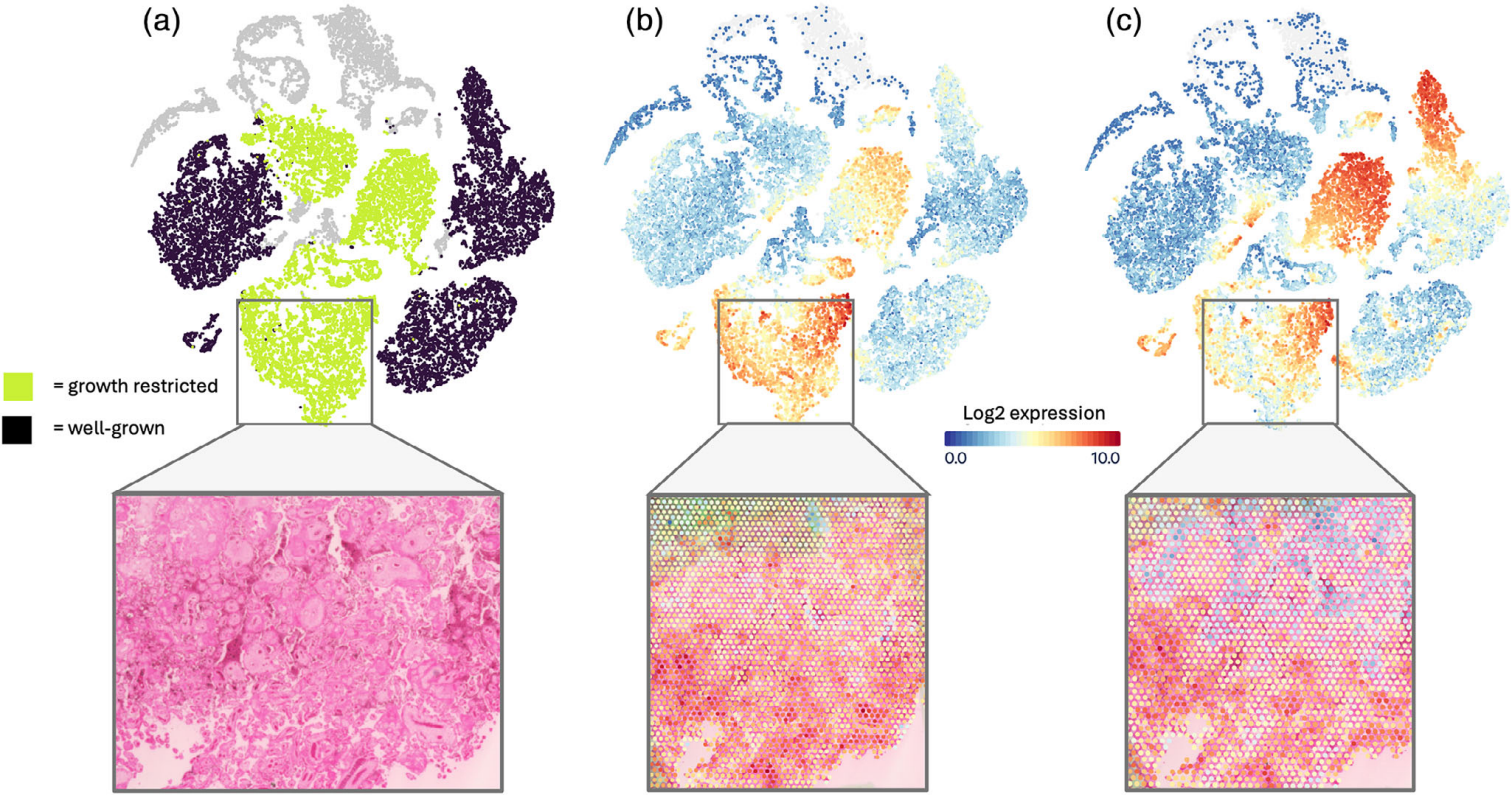
TSNE and representative spatial projections for (A). Clinical characteristics of the sample‐gestational age at delivery highlighted for well‐grown cases; (B) expression of stress‐hormone–associated transcripts; (C) expression of placental interface‐modulating transcripts. TSNE plots provide a visual representation of high‐dimensional data reduced to fewer dimensions for the purpose of visualization while preserving local structure and relationships. Spots near each other represent areas of similar transcriptional pathology.

After processing to remove poor quality or acellular spots, a total of 10,043 capture spots remained from placentas associated with FGR and 12,030 from those without FGR. Unsupervised clustering and resultant t‐distributed stochastic neighbor embedding plots (TSNE plots, see Figure 3) demonstrated relative likeness of the spots from FGR placentas.

**FIGURE 4:**
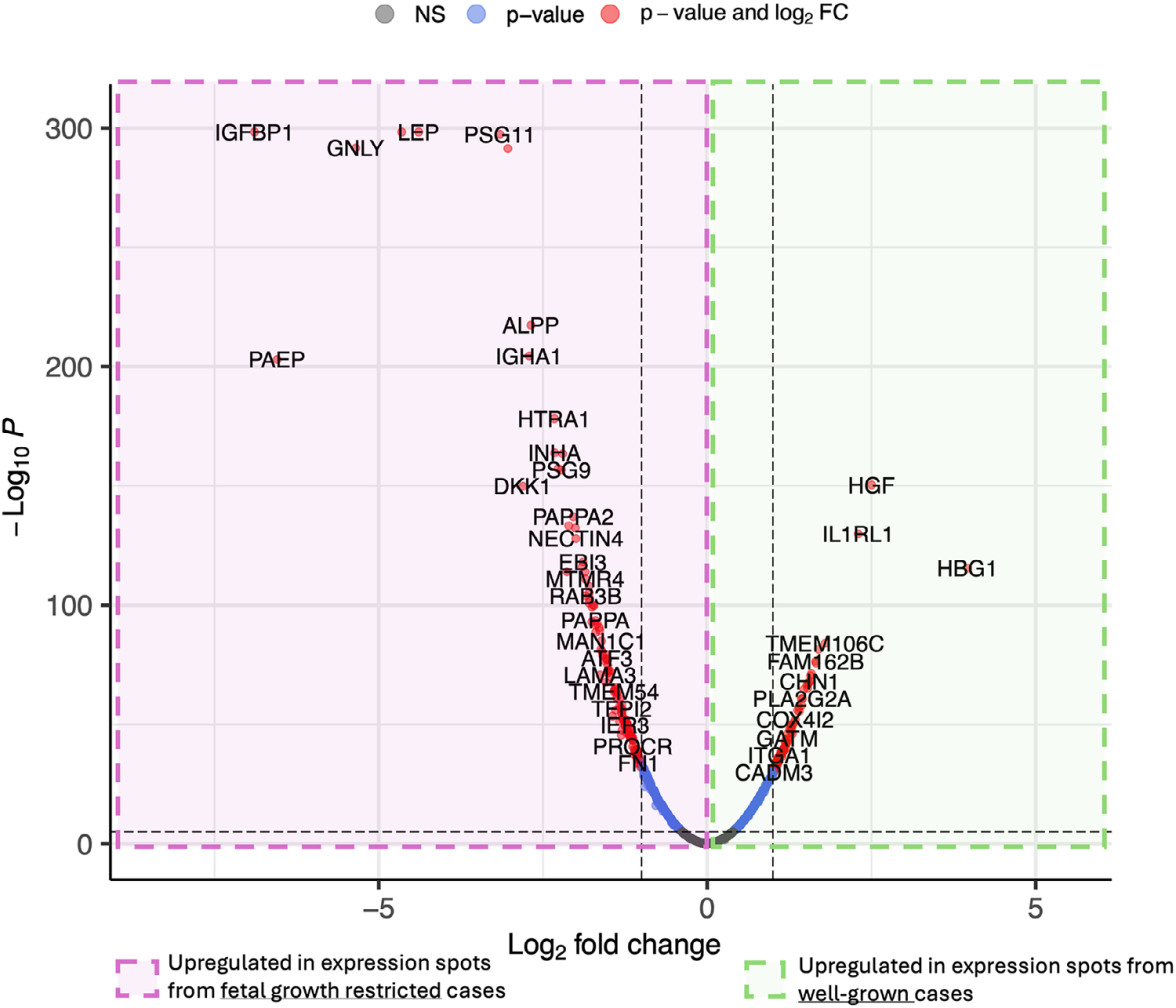
Volcano plot. A volcano plot of significantly differentially expressed transcripts across spatially aligned RNA expression spots from well‐grown and growth‐restricted cases. Volcano plots help to visualize the relationship between statistical significance and the magnitude of change. Genes in the pink box represent upregulated genes from FGR cases, whereas spots in the green boxes represent upregulated genes from well‐grown cases. The higher the spot is the larger the magnitude of significance. NS, non‐significant.

In the analysis of spatially aligned RNA expression spots from the placentas with and without FGR, 1473 genes were differentially expressed (*p* < 0.05, Figure 3). Of the total 1473 differentially expressed genes, 746 were upregulated in expression spots from FGR placentas compared to non‐FGR placentas, while 727 were downregulated in spots from FGR placentas relative to non‐FGR placentas based on Log2Fold change. Genes that were most highly upregulated in FGR include IGFBP1, PAEP, GNLY, CRH, and LEP, whereas genes most strongly downregulated in FGR subjects include HGB1, HGF, IL1RL1, TMEM106C, and C7 (see Figure 4). The most highly upregulated genes involved multiple transcripts involved in hormone‐mediated stress responses (FOS, FOB, CRH, LEP, SFRP1, and IGFB1). These transcripts are disproportionately represented in capture spots associated with FGR placentas (Figure 3B). By contrast, key modulators of the placental interface that are implicated in placental attachment, angiogenesis, and maintenance of fetal immune tolerance are relatively upregulated in placental capture spots from pregnancies diagnosed with FGR (PAEP, INHA, PAPPA2; see Figure 3C). The overall contrasts between up and downregulated transcripts with respect to FGR are shown in Figure 4.

### 3.4 Gene set enrichment analysis and gene ontologies

**FIGURE 5:**
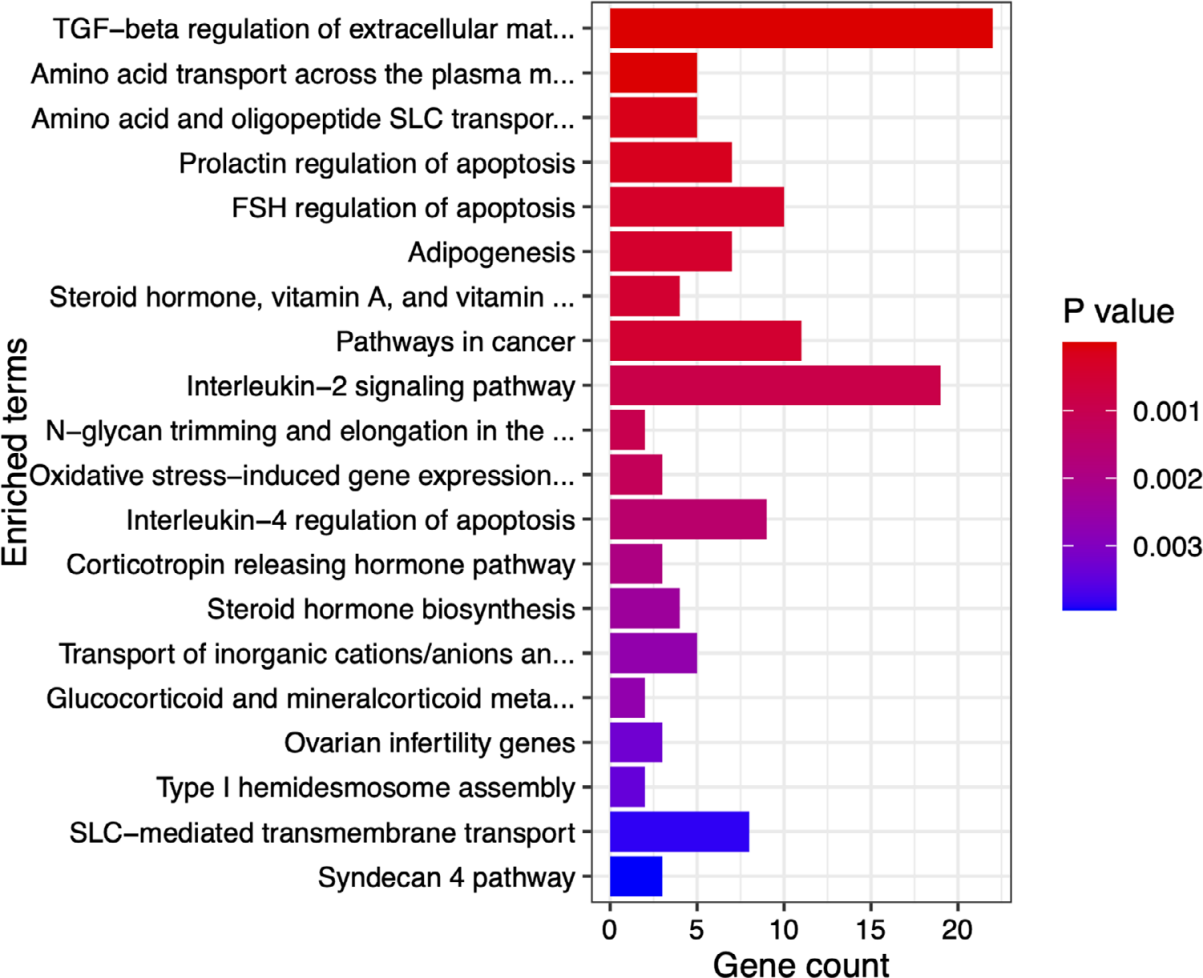
Gene set enrichment analysis of the top 50 upregulated genes in FGR spots. Longer pathway names are abbreviated, see the Bioplanet 2019 term library for the entire pathway description.

Gene set enrichment of the placental spots associated with FGR further highlighted upregulation immune system and inflammation‐mediated pathways involved in the maintenance and remodeling of the placental interface. TGF‐beta regulation of the extracellular matrix, for example (see Figure 5), was a highly significant upregulated pathway in FGR spots specifically, along with interleukin‐2 signaling. Pathways related to stress hormone response, such as the corticotropin‐releasing hormone pathway, are also noted to be significant in this analysis, mirroring the thematic groupings identified through inspection of the most differentially expressed transcripts in FGR spots (Figure 4).

**FIGURE 6:**
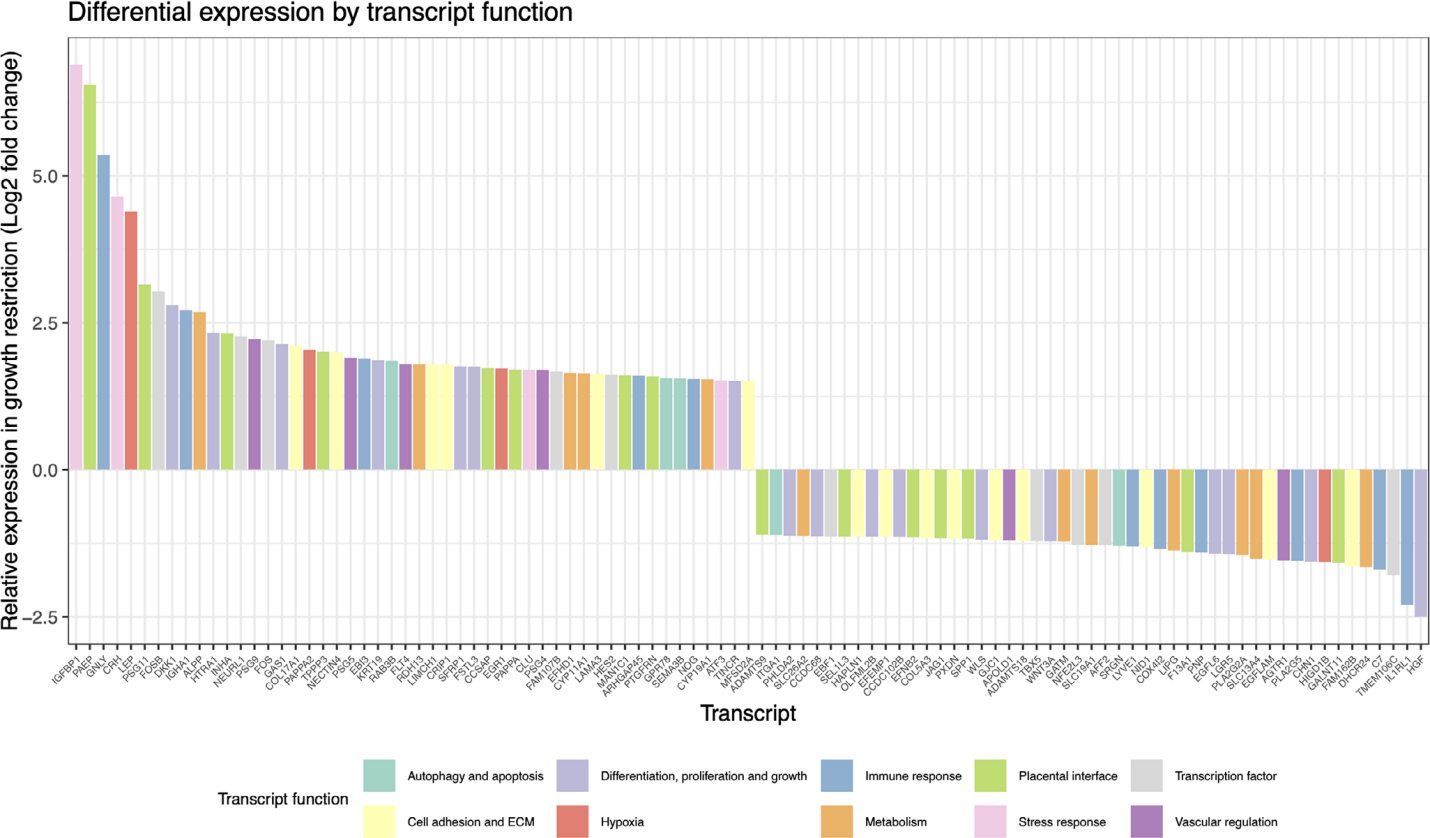
Categories of transcript function on manual review of the top 50 upregulated and downregulated genes in placental capture spots associated with clinically diagnosed fetal growth restriction. ECM, extracellular matrix.

Manual review of the top 50 upregulated and downregulated transcripts in FGR‐associated placental spots versus non‐FGR spots recapitulated the findings of stress hormone and placental interface transcript upregulation in FGR with the complementary observation that transcripts with functions in growth, differentiation, and immune response are also downregulated in FGR‐associated spots (see Figure 6). Transcripts with functions categorized as pertaining to metabolism and cell‐adhesion/the extracellular matrix were identified in the top differentially expressed genes as well. Hypoxia and vascular regulation transcripts were also noted, but less prevalent within the top 50 upregulated/downregulated transcript sets.

## 4 DISCUSSION

In this study, we investigated the molecular mechanisms underlying obstetrical complications seen in pregnancies of individuals with single‐ventricle physiology after Fontan palliation. We applied spatial transcriptomic analysis to a set of clinically characterized, histopathologically reviewed placentas to identify key contrasts in those diagnosed with fetal growth restriction versus those with well‐grown fetuses. A high rate of obstetric complications is observed in our sample of pregnancies from patients with Fontan circulations, as has been well‐documented in the literature [1, 2]. Our work highlights the extensive placental pathology observed in concert with these clinical outcomes, pointing to the placenta as a likely mediator of fetal growth restriction seen in such cases. In our transcriptomic analysis which is co‐located to demonstrable areas of placental pathology, we identify stress hormone response, inflammation/immune activation, modulation of the placental interface, and cell adhesion/extracellular matrix remodeling as involved pathways in FGR seen in pregnancies complicated by maternal Fontan circulation.

FGR is thought to be a state driven by placental insufficiency. In patients with Fontan circulation, this may be secondary to reduced delivery of oxygen and nutrients through the placenta and/ or the Fontan circulation associated chronic venous hypertension, low cardiac output with limited capacity to augment, and relative hypoxia. FGR pregnancies in our data showed marked elevations in terminal pathway upregulation from many genes previously linked to hypoxia and stress. FOS has previously been associated with control of trophoblast migration and invasion [21]. It is also upregulated in instances of late‐term hypoxia in mice [22]. Our data demonstrate that both FOS and FOSB were significantly upregulated in Fontan placentas with FGR. Additional enriched hypoxia‐associated genes included LEP and EGR1. LEP, or leptin, is an endocrine hormone that regulates energy homeostasis. This gene has been tied to FGR, possibly as a compensatory mechanism in instances of uteroplacental insufficiency and inadequate oxygen transfer [23]. Metabolic pathways were also predominantly affected in transcriptomic studies of liver biopsies from FALD [10, 11]. Though the specific transcripts affected differ across tissue types, thematic similarities, such as involvement of the cytochrome P450 pathway, are notable. Likewise, INHA was upregulated in FGR placentas in this study and in single cell transcriptomics of FALD cases [11]. These shared transcripts suggest some molecular overlap between extracardiac manifestations of Fontan circulation.

With respect to placenta‐specific pathology, many of the genes identified in our study have previously been reported in disease states of abnormally shallow placentation. IGFBP1 (insulin‐like growth factor binding‐protein 1), which was strongly enriched in our FGR cohort, has been implicated in growth‐restricted states attributed to hypoxia and fetal stress. Mechanistically, IGFBP1 is known to decrease the activity of insulin‐like growth factor 1, a crucial growth stimulator in the fetus, thought to be a driver of FGR [24]. In the case of PAPPA and PAPPA2, both have been associated with preeclampsia and HELLP syndrome, though there is conflicting data about their role and direction of enrichment in the literature. Both genes drive insulin‐like growth factor‐binding protein protease cleavage, with some models suggesting that they are upregulated as a consequence of hypoxia [25, 26]. Other studies show reduced levels of PAPPA and PAPPA2 in FGR using placental qPCR [27]. The conflicting data may be due in part to sample timing during pregnancy, with low levels of PAPPA being potentially indicative of pathologic insufficiency and higher levels representing an adaptation to stress. Our data from Fontan placentas at the time of delivery demonstrate upregulation of both PAPPA and PAPPA2, likening our growth‐restricted samples to early onset preeclampsia with evidence of hypoxia [26]. Similarly, our finding of upregulated INHA, the transcript encoding inhibin A, aligns with prior transcriptomic analyses of placentas from chronic hypertensive pregnancies [12].

As observed in that work on hypertensive pregnancies, transcripts related vascular function that are not specific to placental tissue are dysregulated in placental syndromes. In the setting of morbidly adherent placentation however, the directionalities of these differences contrast with our results. HGF (Hepatocyte growth factor), for example, is relatively upregulated in a single‐cell transcriptomic study of morbidly adherent placentas [28], but relatively downregulated in growth‐restricted cases within our data. IL1RL1exhibits a similar inverse relationship in invasive placentation [28] versus our growth‐restricted cases. Collectively, these observations highlight similarities between Fontan‐associated placental pathology and placental syndromes attributed to an underdeveloped, dysfunctional placental interface. They point to transcripts related to vascular development that are relatively placenta‐specific (PAPPA/PAPPA2 and INHA) as well as those more generally associated with the endothelium (e.g., HGF) and immune regulation (e.g. IL1RL1) as markers of poor outcomes in placental syndromes that can be applied in Fontan circulation.

Irrespective of the specific transcripts, our results highlight the shared features of growth restriction in the setting of Fontan circulation and pregnancies complicated by hypertensive disorders of pregnancy, such as preeclampsia. The downstream consequence of abnormal hemodynamic and vascular physiology may be driving many of these transcriptional patterns as a compensatory mechanism, resulting in common clinical features. FGR is a common complication of both preeclampsia and Fontan physiology and, like early onset preeclampsia [29], FGR in congenital heart disease is associated with cardiac events [30]. A recent large‐scale review of clinical outcomes of 255 Fontan pregnancies, only reports one case of preeclampsia [1], much lower than the general population's average rate of preeclampsia development. This relative mutual exclusivity deserves further investigation as it could suggest shared mechanisms with different maternal phenotypes.

The transcriptome represented in RNA sequencing studies—with or without spatial localization—does not necessarily mirror the proteome, as translation and post‐translational modifications can alter protein products. Nonetheless, the transcripts identified in this work are candidates for further targeted study, either in cell‐free RNA or as proteins. Though our results have the inherent limitation of being derived from delivered specimens, markers of placental dysfunction have previously been identified in maternal serum prior to delivery [31]. The placentas studied here were all delivered preterm, suggesting that these changes are actively taking place through the early third trimester. In addition to validating our findings in larger throughput methodology and in the proteome, future work should attempt to establish a timeline for differentiating these placental markers in well‐grown and growth‐restricted fetuses of patients with single‐ventricle physiology.

### 4.1 Limitations and future directions

The level of detailed, histopathologic resolution afforded by spatial transcriptomics inherently limits its generalizability. Sample sizes in studies utilizing this technique are typically small, because even with a larger sample, the observations made in a spatially co‐located approach are nonetheless restricted to the pathology isolated in the sample set. An additional limitation, as referenced above, is that these samples are collected at the time of delivery. Transcriptomic placental data does not necessarily correlate to serum studies. As such, any candidate genes identified would have to be confirmed with additional serum studies to identify biomarker candidacy. This study is intended to be hypothesis‐generating and may serve as the foundation for larger studies that explore novel biomarkers or more targeted experiments in model systems. Based on the work presented here, stress hormones such as corticotropin‐releasing hormone or leptin could be studied as potential early indicators of clinically relevant placental pathology as candidate serum markers of maternal‐fetal decompensation in pregnancies complicated by Fontan circulation. Similarities with HDPs should also prompt comparative placental analyses and the consideration of modeling placental stress across these clinically distinct settings.

## 5 CONCLUSION

In summary, this study provides the first of its kind description of placentas from people with Fontan circulation using spatial transcriptomics. It identifies modulation of stress hormones and immune‐mediated remodeling at the placental interface as novel candidate processes for explaining the high rates of obstetric morbidity in this condition. Additional work is needed to define the role of these transcript targets, either as causes or effects in the complex interplay of placental adaptation. Studies should be performed to test the clinical applications of the transcripts identified here for fetal surveillance in pregnancies affected by single‐ventricle physiology. Furthermore, assessment of the generalizability and comparability of these findings between other clinical scenarios with placentally medicated obstetrical complications is needed. Nonetheless, the current work represents a crucial first step toward understanding the way in which single‐ventricle physiology relates to the observation of placental pathology.

## CONFLICT OF INTEREST STATEMENT

The authors declare no conflicts of interest.

## FUNDING STATEMENT

Funding was provided by the Strecher Family Fellow Award through the Department of Pediatric Cardiology at the University of Michigan.

## ETHICS STATEMENT

This study was exempted by the Institutional Review Board of the University of Michigan (IRB# HUM00244157) and follows standard ethical principles.

## Data Availability

The data that support the findings of this study are openly available in Gene Expression Omnibus at https://www.ncbi.nlm.nih.gov/geo, reference number GSE303933.
